# A Systematic Review of Interventions That Reduce Family and Friend Caregiving Time

**DOI:** 10.1093/geroni/igaa057.1126

**Published:** 2020-12-16

**Authors:** Zachary Baker, Eric Jutkowitz, Joseph Gaugler

**Affiliations:** 1 University of Minnesota, Minneapolis, Minnesota, United States; 2 Brown University, Providence, Rhode Island, United States

## Abstract

The decreasing number of family/friend caregivers available to help the rising number of older adults is creating a critical family care gap. For this reason, there is a growing need for interventions that reduce family/friend caregiving time. We systematically reviewed five electronic databases to identify randomized trials, case control, quasi-experimental, and cross-sectional studies that evaluated a modifiable element that could be targeted for interventions with care recipients 65+ and/or their family/friend caregivers and reported on an outcome of time spent caregiving. We excluded studies without a comparison, broadly defined. The initial search included 1,812 unique records. Following abstract and title screening 311 full-texts were reviewed. Fifty-five studies published between the years of 1990 and 2019 met inclusion criteria. Studies predominantly focused on care recipients with dementia (58%) and were largely conducted in western countries (91%). The categories of interventions reviewed included pharmaceuticals (25%), public long-term care financing (7%), case management (7%), care setting (16%), technology (7%), multi-component interventions (9%), skills building (15%), additional formal expertise/care (9%), and other (5%), with one study falling into multiple categories. Pharmaceuticals, case management, care setting, and multi-component interventions demonstrated promising evidence to reduce family/friend caregiving time. Methodologically, studies were inconsistent in measurement and ascertainment of caregiving time. Given the public health concerns of reduced availability of family/friend caregivers for older persons in the upcoming decades, caregiving interventions should consider measurements of caregiving time as key outcomes.

